# Genotoxic stress induces Sca‐1‐expressing metastatic mammary cancer cells

**DOI:** 10.1002/1878-0261.12321

**Published:** 2018-06-13

**Authors:** Jianlin Gong, Benjamin J. Lang, Desheng Weng, Takanori Eguchi, Ayesha Murshid, Thiago J. Borges, Sachin Doshi, Baizheng Song, Mary A. Stevenson, Stuart K. Calderwood

**Affiliations:** ^1^ Department of Medicine Boston University Medical Center MA USA; ^2^ Department of Radiation Oncology Beth Israel Deaconess Medical Center Harvard Medical School Boston MA USA

**Keywords:** genotoxic stress, radiation bystander effect, radiation therapy, responses to cancer therapy, Sca‐1

## Abstract

We describe a cell damage‐induced phenotype in mammary carcinoma cells involving acquisition of enhanced migratory and metastatic properties. Induction of this state by radiation required increased activity of the *Ptgs2* gene product cyclooxygenase 2 (Cox2), secretion of its bioactive lipid product prostaglandin E2 (PGE2), and the activity of the PGE2 receptor EP4. Although largely transient, decaying to low levels in a few days to a week, this phenotype was cumulative with damage and levels of cell markers Sca‐1 and ALDH1 increased with treatment dose. The Sca‐1^+^, metastatic phenotype was inhibited by both Cox2 inhibitors and PGE2 receptor antagonists, suggesting novel approaches to radiosensitization.

**Abbreviations**
Aldh: aldehyde dehydrogenase
Cox2: cyclooxygenase‐2
CSC: cancer stem cell
EP4: prostaglandin E receptor 4
GPI‐AP: glycosylphosphatidylinositol‐anchored surface protein
Gy: Gray
ICC: immunocytochemistry
IHC: immunohistochemistry
IR: irradiation
MMT: mucin‐1 MMTV‐PyMT transgenic mice
MMTV: mouse mammary tumor virus
PGE2: prostaglandin‐E2
PyMT: polyomavirus middle T‐antigen
Sca‐1: stem cell antigen‐1
TIC: tumor initiating cell

## 1 Introduction

Cancer cells that survive cytotoxic therapies such as radiation or genotoxic agents can become highly resistant to further treatment and develop an increased capacity for repopulation, invasion, and metastasis (Lee *et al*., 2017; Weng *et al*., 2012; Woodward *et al*., 2007). The underlying mechanisms that lead to such treatment‐induced tumorigenic properties are of clinical importance but not yet fully understood. Our studies showed that such cells expressed high levels of the surface marker Sca‐1 (stem cell antigen‐1, also known as Ly6A). Sca‐1 is a mouse glycosylphosphatidylinositol‐anchored surface protein (GPI‐AP), initially used as a marker of hematopoietic stem cells (SC) (Bradfute *et al*., 2005; Holmes and Stanford, 2007) and more recently some populations of mammary tumor initiating cells (TIC) (Weng *et al*., 2012). Sca‐1 is regulated downstream of Wnt/beta‐catenin signaling and is thought to play a functional role in mammary cancer tumor initiation and cells undergoing a cellular wound healing response, triggered by cell death and migration (Batts *et al*., 2011). Mammary tumor cell populations responding to cytotoxic therapy (Lee *et al*., 2017; Weng *et al*., 2013; Woodward *et al*., 2007) may undergo dedifferentiation to a more resistant state in a fraction of the population. A similar phenomenon was termed the ‘Phoenix Rising’ response and was shown to be triggered through activation of caspases 3 and 7 induction of phospholipase A2 (Huang *et al*., 2011; Li *et al*., 2010).

In this study, we have examined the properties of cells expressing high levels of Sca‐1 after cytotoxic treatment and key mechanisms involved in increased tumor cell migration and metastasis. In previous studies, increased resistance to radiation and enhanced metastasis was found in a population of primary mouse mammary cells expressing cell surface Sca‐1 (Holmes and Stanford, 2007; Weng *et al*., 2012, 2012). In this study, we showed that radiation induces cells (4T1, MMT) expressing high levels of Sca‐1 which exhibit enhanced migratory and metastatic activities. The response required induction of cyclooxygenase 2 (Cox2) and its bioactive lipid product prostaglandin E2 (PGE2). This phenotype was however inhibited by both Cox2 inhibitors and PGE2 receptor antagonists, leading to profound inhibition of radiation‐induced metastasis.

## 2 Results

### 2.1 Enrichment in subpopulations of mammary carcinoma cells expressing elevated surface levels of Sca‐1 after cytotoxic treatment

**Figure 1:**
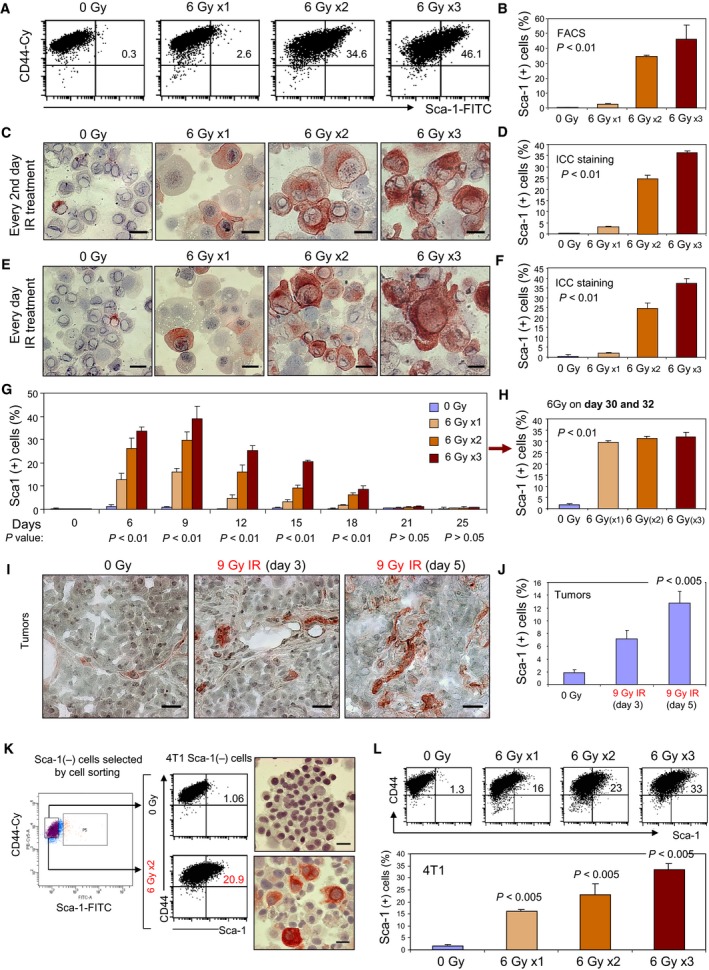
Genotoxic stress leads to enrichment of Sca‐1 in the 4T1 cell line and in MMT mammary tumors. (A) 4T1 cells were irradiated with 6 Gy for up to three times. After IR, cell culture medium was replaced and cells were collected for FACS analysis with the indicated antibodies. (B) The mean percentage of CD44 and Sca‐1 double‐positive cells in three repeated experiments is shown ± SD. (C–F) 4T1 cells were irradiated every second day (C) or every day (E) and Sca‐1 expression assessed by ICC staining, scale bar 20 μm. The mean percentage of Sca‐1‐positive (red‐stained) cells is also shown ± SD (D and F). (G) Timeline of Sca‐1 induction in 4T1 cells irradiated with 6 Gy for one, two, or three times. The cells were collected at the indicated times, stained with Sca‐1 mAb, and analyzed by FACS. (H) Cells treated as in G were irradiated again with 6 Gy twice on day 30 and 32 when Sca‐1 levels had returned to baseline, and then assayed for the re‐expression of Sca‐1 after overnight culture. The *P* values were obtained by re‐irradiated cell to non‐re‐irradiated cells previously irradiated one, two, or three times. (I) MMT tumor cells were transplanted into wild‐type C57BL/6 mammary fat pad as described in Experimental procedures. On day 10, established tumors were treated with 9 Gy radiotherapy. Sca‐1 expression with tumor sections was assessed by IHC on days 3 and 5 after treatment, scale bar 20 μm. (J) Mean numbers of Sca‐1^+^ cells in imaged IHC MMT tumor sections is shown ± SD. (K) 4T1 cells were stained with anti‐CD44 and anti‐Sca‐1 mAbs, and then, Sca‐1^−^ population was selected by cell sorting (left side). Sca‐1^−^ 4T1 tumor cells were then separated into a nonirradiated sample and an irradiated 6 Gy × 2 every other day sample. After ON culture, the cells were stained with anti‐Sca‐1 and‐CD44 mAbs and analyzed by FACS and ICC, scale bar 20 μm. (L) The Sca‐1^−^ population of 4T1 cells were irradiated with 6 Gy once, twice or three times, and then analyzed by FACS. Nonirradiated cells were used as controls. The percentage of Sca‐1^+^ cells is presented in the bar graph.

Surface levels of Sca‐1 were shown previously to be increased in radioresistant, invasive, and metastatic MMT mammary carcinoma cells *in vitro* and *in vivo* (Weng *et al*., 2012). In this study, we used 4T1 murine mammary rather than MMT for most studies as MMT, being primary cells grew for only one to two generations *in vitro*. When 4T1 carcinoma cells were treated with gamma‐irradiation in fractions of 6 Gy for up to three times, the population of cells positive for Sca‐1 was found to increase with escalating dose (Fig. 1A, B) and similar changes were observed using immunocytochemical (ICC) staining (Fig. 1C–F). Irrespective of whether cells were irradiated daily or every second day, similar increases in the numbers of Sca‐1‐positive cells were observed (Fig. 1C–F). Although the degree to which Sca‐1 increases differed in cells irradiated one, two or three times, the kinetics of expression and decay were similar, with significant increases observed on day 6, induction peaking on day 9 and Sca‐1 levels thereafter declining (Fig. 1G). On day 21, the Sca‐1 levels had almost returned to the baseline in each case, although a small number of Sca‐1^+^ cells were retained (Fig. 1G). Interestingly, re‐exposure of previously irradiated cells after decay in Sca‐1 levels, regardless of whether once or three times pretreated, boosted the expression of Sca‐1 (Fig. 1H). Cells thus seemed to have a prolonged molecular memory of previous irradiations in terms of re‐induction of Sca‐1. In addition to radiation treatment, Sca‐1^+^ cells were also induced by the cytotoxic drugs doxorubicin and cisplatin, indicating a general response to genotoxic stress (Fig. S1).

When we examined the effects of tumor irradiation *in vivo*, we observed a similar phenomenon, with cells staining positive for Sca‐1 (Fig. 1I). Significant increases in Sca‐1^+^ cells were observed within tumor sections on day 3, increasing to day 5 (Fig. 1I, J).

We next tested the possibility that enrichment of Sca‐1^+^ cells was due to preferential killing of radiation‐sensitive Sca‐1^−^ cells. To determine the origin of Sca‐1^+^ cells, 4T1 mammary carcinoma cells were sorted to select the Sca‐1^−^ cells (Fig. 1K), and the Sca‐1^−^ cells were then exposed to radiation. Even though these populations were selected to exclude Sca‐1^+^ cells, radiation still led to the expression of Sca‐1, with up to 33% of these cells becoming Sca‐1^+^ after three 6 Gy radiation exposures (Fig. 1K, L). To examine whether the observed enrichment of Sca‐1^+^ cells was due to divergent rates of radiation‐induced cell death between Sca‐1^+^ and Sca‐1^−^ cells, we examined cell viability within each of these populations after treatment. Unlike lymphoid‐derived cancer cells that readily undergo apoptosis after irradiation the fate of most irradiated tumor cells of epithelial origin is senescence, necrosis, or a slow form of death termed mitotic catastrophe. These latter fates of epithelial tumor cells are alternative outcomes to apoptosis, which is largely inaccessible to many types of cancer due to frequent genetic disruptions in the apoptotic pathway. Thus, in most tumor types of epithelial origin, apoptosis is rare. At the dosages used in this study, our analysis showed no statistical difference in cell viability between irradiated and nonirradiated cells within 24 h after irradiation (Fig. S2A). In addition, no statistical difference in cell viability was observed between Sca‐1^+^ and Sca‐1^−^ cells within irradiated samples (Fig. S2B). Together, these data indicate irradiation strongly induces Sca‐1 expression by a mechanism that does not involve selective cell death of Sca‐1^−^ cells.

### 2.2 Irradiation induces Sca‐1‐expressing cells with enhanced migratory and metastatic ability

**Figure 2:**
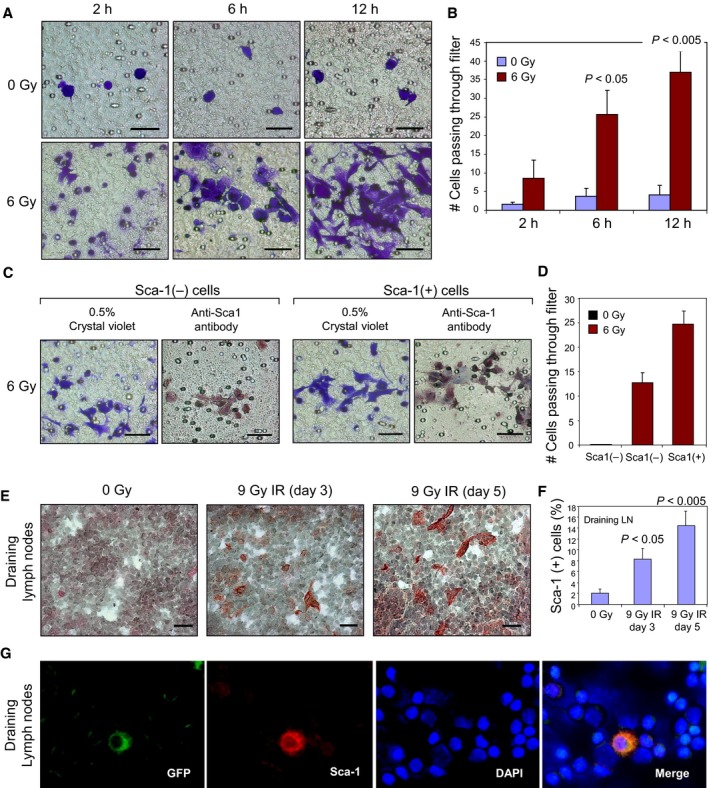
Irradiation induces Sca‐1‐expressing cells with enhanced migratory and metastatic ability. (A) 4T1 cells were irradiated with 6 Gy ×2 and assayed for cell migration using a two‐chamber method. Irradiated or control 4T1 cells were placed in triplets on the filter of upper side of a migration chamber and cultured. At the indicated times, the filters were gently removed and the cells on the upper side of filter were cleared. The cells in the lower side of filter were fixed with 4% paraformaldehyde and stained with 0.5% crystal violet. Filters were then photographed with representative fields shown, scale bar 50 μm. (B) Mean number of migrated cells per field under 10× magnification is shown ± SD. The *P* values were obtained by comparing the mean number of migrated cells between nonirradiated cells (0 Gy) and irradiated cells. (C and D) 4T1 cells were sorted into Sca‐1 (−) or Sca‐1 (+) cells that were then treated with twice 6 Gy every other day. The migration ability of these cells was measured by a two‐chamber method. After 6 h of culture, the cells migrated to the other side of the filter were stained with 0.5% Crystal violet or anti‐Sca‐1 antibody, scale bar 50 μm. The cells were counted and mean cells per field is presented in the bar graph for each condition ± SD. (E) Sca‐1 expression was assessed by IHC staining of draining lymph node sections of nonirradiated MMT tumors and tumors 3 and 5 days after 9 Gy irradiation, scale bar 20 μm. (F) The percentage of Sca‐1‐positive cells in draining lymph nodes is represented ± SD. (G) GFP‐MMT tumor cells within the draining lymph nodes of wild‐type C57BL/6 mice could be detected by GFP expression and were positive for Sca‐1.

Our previous experiments had shown the Sca‐1^+^ MMT mammary tumor cells to have enhanced migratory and metastatic properties (Weng *et al*., 2012). This was true both for the fraction of such cells present in untreated MMT mouse mammary cell populations *in vivo* that mediate cell renewal and the radiation‐induced Sca‐1^+^ fraction (Weng *et al*., 2012; Weng *et al*., 2012). To further determine the properties of the Sca‐1^+^ cells in 4T1 cells, they were exposed to ionizing radiation then assayed for migratory ability, using a double‐chamber method. Irradiation markedly increased the cell number capable of penetrating the filter (Fig. 2A, B). To determine the phenotype of the cells passing through the filter, Sca‐1^+^ and Sca‐1^−^ 4T1 cells were selected by cell sorting, and then twice irradiated with 6 Gy and subjected to cell migration assay. Consistent with the findings in Fig. 2A, B, no cells in the Sca‐1^−^ population passed through the filter if nonirradiated. In contrast, many cells in both irradiated Sca‐1^−^ and Sca‐1^+^ populations migrated through the filter (Fig. 2C, D). Moreover, cells that migrated through the filter were Sca‐1^+^ whether from either sorted Sca‐1^−^ or Sca‐1^+^ populations (Fig. 2C, D). The absolute numbers of cells passing through the filter in the irradiated Sca‐1^+^ cells were more than those in the irradiated Sca‐1^−^ cells (Fig. 2C, D). These results indicate Sca‐1^+^ cells, regardless of their origin from Sca‐1^+^ or Sca‐1^−^ cell populations, possess enhanced migratory ability. We next examined the effects of radiation on MMT tumor metastasis *in vivo*. We chose this mouse mammary tumor model as MMT cells have been engineered to express GFP and can thus be positively identified and distinguished from normal cells in metastatic tissues. ICC staining of sections of primary MMT tumors growing in the mammary fat pads had shown marked enrichment for Sca‐1 after radiation treatment (Fig. 1I). Analysis of the draining lymph nodes from the same animals indicated marked increases in Sca‐1‐stained cells (Fig. 2E, F). These findings therefore suggested increased metastasis of the Sca‐1^+^ cells after radiotherapy. Metastasis from the primary site was confirmed by detection of GFP‐expressing tumor cells in the lymph nodes after irradiation of the primary site that also stained positive for Sca‐1 (Fig. 2G).

### 2.3 Changes in gene expression after radiotherapy

To further define the phenotypes of Sca‐1^+^ cells produced in response to radiation treatment, we next examined the relative expression of a subset of mRNAs in irradiated tumor cells using a qPCR array specific for TIC‐associated genes (Fig. S3A). The mRNA species that were up‐ or downregulated in 4T1 cells in response to radiation are therefore shown in Fig. S3A. Genes upregulated in irradiated 4T1 cells included *Aldh1a1* (Nozaki *et al*., 2017) and inducible nitric oxide synthase *Nos2* (Kim *et al*., 2013) known to be increased in TIC, Pecam‐1, Lin28A, and Ptprc/CD45 which participate in cancer and embryonic stemness (Lin *et al*., 2014; Shyh‐Chang and Daley, 2013; Williamson *et al*., 2013), luminal progenitor marker c‐Kit (Visvader and Stingl, 2014), and ABCG2, a protein involved in SC chemoresistence (An and Ongkeko, 2009). Real‐time RT‐qPCR confirmed large increases in *Sca‐1* and *Aldh1a1* mRNA (Fig. S3B). Of note, *Aldh1a1* was upregulated 10‐ to 12‐fold compared with levels in control cells (Fig. S3). These data were also confirmed by the finding that ALDH enzymatic activity was significantly increased in the irradiated murine 4T1 (Fig. S4A, B) and human MCF7 mammary carcinoma cells (Fig. S4C, D). In addition, the ALDH‐enriched 4T1 population also had a Sca‐1^+^ phenotype as determined using a triple staining/flow cytometry approach (Fig. S4E). Exposure to radiation thus led to a profound increase in the subpopulation of cells with elevated levels of surface Sca‐1 and intracellular ALDH1.

### 2.4 The Sca‐1^+^/ALDH1^+^/migratory phenotype was induced using medium conditioned by irradiated cancer cells

**Figure 3:**
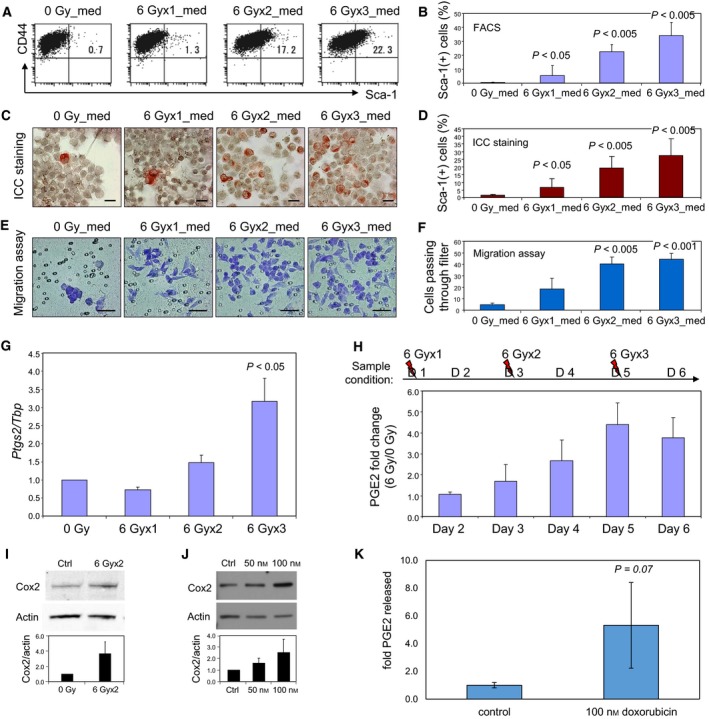
Medium conditioned by cells exposed to genotoxic agents induces elevated Sca‐1 levels and increased migration in nonirradiated cells. 4T1 cells were cultured with medium derived from 4T1 cells irradiated with 6 Gy one, two, or three times. The radiation‐conditioned medium (med) was changed every other day. (A) 4T1 cells cultured with medium derived from irradiated cells were stained with the indicated mAbs and analyzed by FACS. (C) Cells treated as in A were visualized with anti‐Sca‐1 antibodies, using ICC staining, scale bar 20 μm. The mean percentages of Sca‐1‐positive cells in three experiments are shown ± SD (B, D). Cell migration assays were next carried out on cells exposed to conditioned medium, using a two‐chamber method (E, F). Cells treated as in A and C were placed in triplet wells on the upper side of the filter and incubated for 12 h. The cells in the lower side of the chamber were then stained and counted, scale bar 50 μm (E). Representation of mean across three replicate experiments is shown ± SD;* P* values were obtained by comparing cells cultured with medium from nonirradiated cells with those obtained from one, two, or three times irradiated cells (F). (G) *Ptgs2* mRNA expression 24 h post‐IR measured by real‐time qPCR normalized to *Tbp*. Expression is represented as ratio to nonirradiated sample. *P* value estimated by one‐way *t‐*test of compiled fold change values to control. Figure shows representative mean of at least three independent experiments ± SD. (H) PGE2 levels in medium from irradiated or nonirradiated cells. Medium was collected at days 2–6 (D1–6) and quantified for PGE2 levels by ELISA. Compiled mean 6 Gy/0 Gy PGE2 ratios from at least three independent experiments are represented ± SEM. (I) Cox2 protein expression analysis by western blot 24 h after a second 6 Gy irradiation. Densitometry‐quantified Cox2 levels were normalized to actin expression and shown as the mean of three independent experiments ± SD. (J) Western blot analysis of Cox2 protein levels after 100 nm doxorubicin for 48 h. Densitometry‐quantified Cox2 levels were normalized to actin expression and shown as the mean of three independent experiments ± SD. (K) 4T1 cells were treated with 100 nm doxorubicin for 48 h; growth media were then replaced, and PGE2 levels in the culture media were assayed by ELISA following 6 h of further culture. Mean fold difference in PGE2 levels is shown across three replicate wells for each condition normalized to cell number within respective well ± SD.

As secreted products can mediate radiation resistance, we next asked whether induction of the Sca‐1^+^CD44^+^ALDH1^+^ migratory phenotype could similarly be caused by factor(s) released from irradiated cells (Brocard *et al*., 2015; Dent *et al*., 2003). Tissue culture medium conditioned by irradiated cells was indeed able to cause formation of a large population of Sca‐1^+^ 4T1 cells when added to previously untreated cultures (Fig. 3A, B). Unlike Sca‐1^+^ cells in irradiated cultures, most of which had an abnormal cellular morphology and multiple and/or giant nuclei (see Fig. 1C, E), cells treated with radiation‐conditioned medium maintained similar nuclear morphology and size to controls (Fig. 3C). In addition, cells cultured in radiation‐conditioned medium acquired enhanced migratory properties proportionate to the dosage of radiation applied to cells from which the medium was taken (Fig. 3E, F). Therefore, bioactive substance(s) appear to be released from irradiated cells mediating the induction of the Sca‐1^+^/migratory phenotype.

One secreted product that has been associated with responses to tissue damage is PGE2 (Li *et al*., 2010). Therefore, we considered the potential role of Cox2 a key enzyme required for PGE2 synthesis in the phenotype. Radiation injury of 4T1 cells led to increased Cox2 mRNA (Fig. 3G) and protein expression (Fig. 3I). In addition, the levels of PGE2 detected in the medium of irradiated 4T1 cells were increased in a dose‐dependent manner (Fig. 3H). Doxorubicin treatment also induced amplification of Cox2 at the protein level and PGE2 production (Fig. 3J, K). Thus, PGE2 is released abundantly from irradiated or doxorubicin‐treated cells and could be one mediator of the cell damage‐induced Sca‐1^+^/migratory phenotype.

### 2.5 Suppression of radiotherapy‐induced Sca‐1^+^ by pretreatment with Cox2 inhibitors

**Figure 4:**
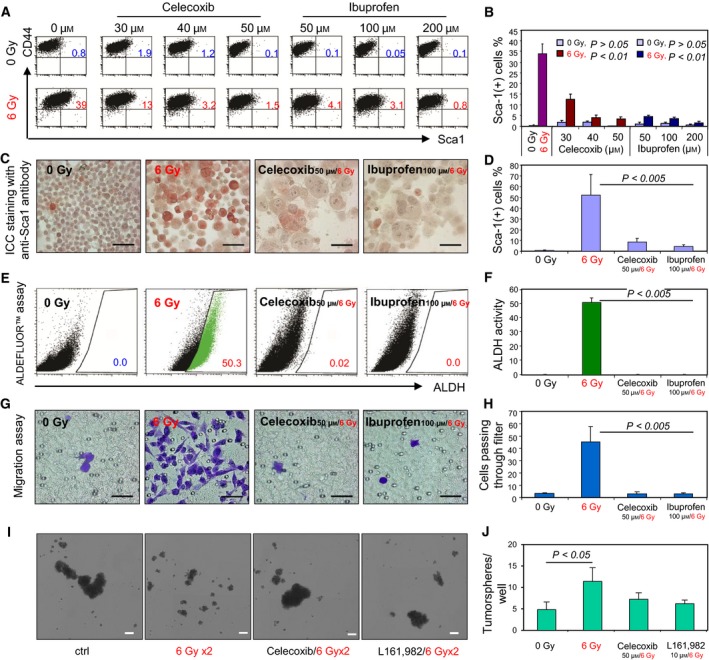
Suppression of IR‐induced Sca‐1 induction by Cox2 inhibitors. (A,B) 4T1 cells were preincubated with the indicated doses of celecoxib or ibuprofen overnight and then irradiated with 6 Gy for up to three times every other day. Nonirradiated 4T1 cells treated with the inhibitors acted as controls. On day 6, 4T1 cells were collected, stained with the indicated antibodies, and analyzed by FACS. Mean percentages of CD44 and Sca‐1 double‐positive cells across three experiments are shown ± SD (B). (C) ICC staining of cells pretreated with 50 μm celecoxib or 100 μm ibuprofen and then irradiated with 6 Gy for up to three times and stained with anti‐Sca‐1 antibodies, scale bar 50 μm. (D) The red Sca‐1‐positive cells (60×) were counted, and the percentage of Sca‐1‐positive cells in three replicate experiments is presented in the bar graph ± SD. (E) ALDH activity was assessed by ALDEFLUOR™ assay in 4T1 samples pretreated with 50 μm celecoxib or 100 μm ibuprofen and irradiated with 6 Gy up to three times. On day 6, ALDH activity was assessed. DEAB‐treated groups were used as respective negative controls. The gated cells with green color indicate cells positive for ALDH activity. (F) The percentage of cells positive for ALDH in three experiments is presented ± SD. (G) Migratory potential of cells pretreated with 50 μm celecoxib or 100 μm ibuprofen and irradiated with 6 Gy up to three times was assessed by the transwell migration assay. Representative images of counted fields are shown, scale bar 50 μm. (H) Cells passing through the filter were counted at 40× magnification. Mean values representative of three experiments are shown ± SD. The *P* values were obtained by comparing irradiated cells without pretreatment of inhibitors with inhibitor‐treated cells at indicated doses. (I) 4T1 cells were assayed for tumorsphere formation in a 24‐well ultra‐low attachment plate after the indicated treatments. Tumorspheres were imaged and quantified after seven days of culture as described in methods. Scale bar indicates 200 μm. (J) Mean tumorsphere counts across quintuple wells for each condition is represented ± SD,* n* = 2.

To assess the role of Cox2 in the radiation‐induced Sca‐1^+^ phenotype, we used the specific Cox2 inhibitor celecoxib (McCormack, 2011). 4T1 tumor cells were pretreated with celecoxib and irradiated with 6 Gy for up to three times. Co‐treatment with celecoxib was effective in blocking induction of Sca‐1^+^ by radiation (Fig. 4A, B). Similar results were obtained in cells pretreated with ibuprofen, a nonsteroidal inhibitor of both Cox1 and Cox2 (Davies, 1998) (Fig. 4A, B). Pretreatment with 50 μm of celecoxib or 100 μm ibuprofen reduced the Sca‐1^+^ cell numbers almost to background levels in radiation‐treated cells (Fig. 4C, D). Similarly, pretreatment with celecoxib or ibuprofen led to a reduction in the cell population with ALDH activity measured by ALDEFLUOR™ assay (Fig. 4E, F). The Sca‐1^+^ fraction of irradiated cells was closely associated with an enhanced migratory and metastatic phenotype (Fig. 2) an effect that was almost completely inhibited by 50 μm celecoxib or 100 μm ibuprofen (Fig. 3G, H). These data provided further evidence supporting the requirement for elevated levels of cellular Cox2 and extracellular PGE2 in the genotoxic treatment‐induced migratory, Sca‐1^+^ cell phenotype. As the cells appeared to be undergoing some level of dedifferentiation after radiation, we also examined tumorsphere formation, an index of tumor stem cell or progenitor properties. Radiation increased tumorsphere formation and this effect was partly inhibited by Cox2 inhibitors or antagonist of the PGE2 receptor EP4 (Fig. 4I, J).

### 2.6 The PGE2 receptor EP4 supports the genotoxic stress‐induced Sca‐1^+^ phenotype

**Figure 5:**
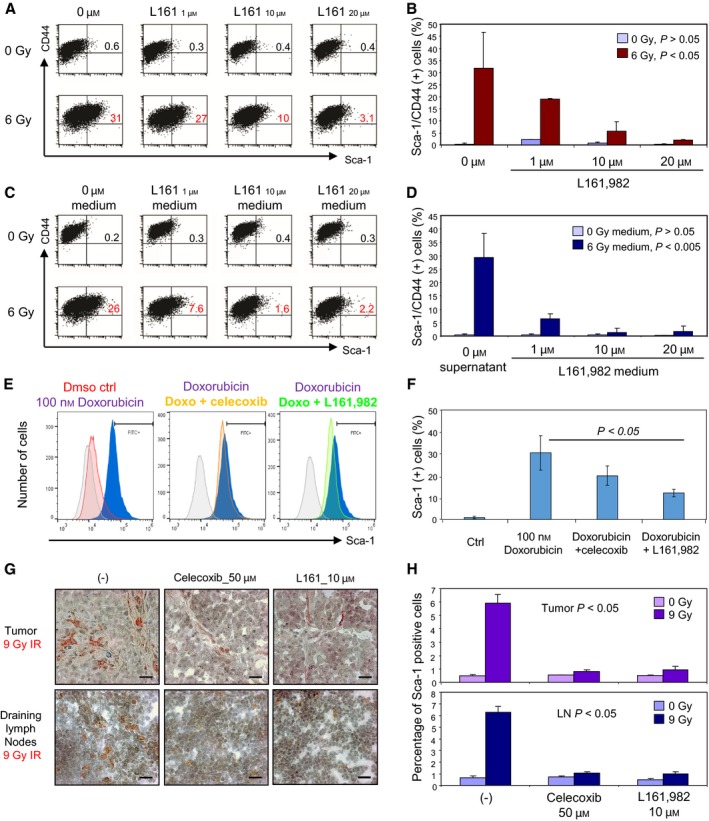
Inhibition of the cytoxan induction of Sca‐1 by PGE2 receptor antagonists. (A) 4T1 cells were preincubated with the indicated doses of L161,892 overnight and then irradiated with 6 Gy three times every other day. Nonirradiated 4T1 cells served as the controls. On day 6, 4T1 cells were collected, stained with indicated mAbs, and analyzed by FACS. (B) Mean percentages of CD44 and Sca‐1 double‐positive cells across three experiments are shown ± SD. The *P* values were obtained by comparing irradiated cells treated without or with inhibitor at indicated doses. (C) 4T1 cells were cultured for 5 days with medium derived from the groups in A. The medium was changed every second day. 4T1 cells were stained with the indicated mAbs and analyzed by FACS. (D) Mean percentages of CD44 and Sca‐1 double‐positive cells across three experiments are shown ± SD. The *P* values were obtained by comparing cells cultured in conditioned medium to cells cotreated with conditioned medium and L161,982 at the indicated doses. (E) 4T1 Sca‐1 surface expression was measured by flow cytometry for cells treated with 100 nm doxorubicin and with or without 40 μm celecoxib or 20 μm L161,982 for 48 h. (F) Mean percentage of Sca‐1‐positive cells after indicated treatments ± SD. Representative experiment shown, *n* = 5. (G) Sca‐1 expression within MMT tumors and draining lymph nodes was assessed *in vivo* by IHC after irradiation (IR) of tumor cells pretreated with or without indicated concentrations of celecoxib or L161,982, scale bar 20 μm. (H) Quantification of Sca‐1^+^ cells within irradiated and nonirradiated MMT tumors (above) and draining lymph nodes (below) is shown, representative of triplicate assays ± SD.

We next asked whether the PGE2 receptor EP4 on the 4T1 cell surface was involved in the induction of Sca‐1^+^CD44^+^ (Fig. 5). Although cells can potentially express at least four PGE2 receptor subtypes (EP1‐4), EP4 seems particularly significant in mammary cancer metastasis (Kundu *et al*., 2009; Ma *et al*., 2006; Reader *et al*., 2011). Blocking EP4 with its specific antagonist L161,982 led to a drug dose‐dependent reduction in the formation of radiation‐induced Sca‐1^+^ cells (Fig. 5A, B). The EP4 inhibitor also suppressed the effects of radiation‐conditioned medium on upregulation of Sca‐1^+^CD44^+^ in 4T1 cells (Fig. 5C, D). Celecoxib and L161,982 also prevented the increases in the Sca‐1^+^ population induced by doxorubicin treatment (Fig. 4E, F). The cumulative results from these studies (Figs 1, 2, 3, 4, 5) therefore suggested a cell damage‐induced programming pathway for this Sca‐1^+^/metastatic phenotype with steps including: Tumor cell injury > Cox2 > PGE2 > PGE2‐R > metastatic Sca‐1^+^ cells.

### 2.7 Effectiveness of Cox2 inhibitors and EP4 antagonists in reducing radiation‐induced tumor metastasis

We next examined the role of Cox2 and PGE2 in the induction of Sca‐1 expression and tumor metastasis *in vivo* in the MMT mouse tumor model (Fig. 5G, H). MMT cells were pretreated with celecoxib (50 μm) or L161,982 (10 μm) overnight, and then, 10^6^ of such cells were inoculated into cleared mammary fat pads. As before, radiation led to increases in Sca‐1^+^ cells in tumors and lymph node metastases (Fig. 5G, H, left panels). However, these effects of tumor irradiation were potently inhibited by the Cox2 inhibitor celecoxib and the EP4 antagonist L161,982 (Fig. 5G, H, middle and right panels).

## 3 Discussion

Our data therefore pointed to a mechanism of dose‐dependent, cytotoxic treatment‐induced induction of mammary carcinoma cells with enhanced migratory, metastatic, and treatment resistant properties (Figs 1 and 2, Fig. S1). Cells and tumors treated with cytotoxic therapies exhibited a large increase in surface Sca‐1^+^, enhanced migration *in vitro*, and increased metastasis. Increases in Sca‐1 levels and migratory potential also correlated with ALDH activity in 4T1 cells, and equivalent radiation treatments also induced ALDH activity in human MCF7 breast cancer cells (Fig. S4). The cluster of properties observed in the Sca‐1^+^ALDH1^+^ cells are sometimes observed in cancer stem cells (CSC) (Marcato *et al*., 2011), and populations sorted for this CSC phenotype typically possess highly invasive and metastatic properties (Weng *et al*., 2012). However, although irradiated cells showed some increases in properties ascribed to CSC (Sca‐1 induction, ALDH expression, tumorsphere formation), it is not clear to what degree the cells correspond to known populations of normal mammary SC. It may be that cell populations exposed to the cytotoxic treatments respond in a homeostatic manner by acquiring some stem‐like properties through dedifferentiation of progenitor or differentiated mammary tumor cells. The two genes observed to be most upregulated in irradiated 4T1 cells were ALDH1 and Sca‐1 itself (Fig. S3). In addition to being stem cell markers, ALDH family proteins play functional roles, protecting stem cells from cytotoxic agents (Ma and Allan, 2011; Tomita *et al*., 2016). Sca‐1 is a surface GPI‐AP, localizing to lipid rafts in cancer cells, and may play a key role in regulating cell migration through signaling mechanisms including modulation of Src family kinase activity (Batts *et al*., 2011; Holmes and Stanford, 2007). It has also been suggested that a Sca‐1 ligand may exist (Holmes and Stanford, 2007), and it would be tempting to speculate that such a ligand could contribute to the effects of radiation‐conditioned medium on cell migration. Irradiated cells also underwent a large increase in c‐kit mRNA (Fig. S3), an important factor in hematopoietic stem cell expansion (O'Laughlin‐Bunner *et al*., 2001). Thus, further expression profiling of the Sca‐1‐rich fraction of irradiated cells would be desirable to expand our understanding of mechanism.

The finding that exposure to conditioned medium from irradiated cells could induce Sca‐1 surface expression and increase cell migration in cells indicates that products secreted into the medium were mainly responsible for inducing this phenotype. Consistent with past reports, we found increased Cox2 activity and PGE2 production to be important factors for the Sca‐1^+^ phenotype, further underlining the importance of PGE2 in cell damage‐induced regenerative responses (Allen *et al*., 2015; Huang *et al*., 2011). PGE2 is thought to be involved in triggering the Wnt‐beta‐catenin pathway known to be activated by irradiation and leading to radiation‐induced stemness (Goessling *et al*., 2009; Woodward *et al*., 2007). In addition, Cox2 expression and PGE2 release have been shown to be dependent on activation of the transcription factor NFkB (Nakao *et al*., 2002), which itself is induced by ionizing radiation (Dent *et al*., 2003; Huang *et al*., 2011; Wang *et al*., 1996). The findings that radiation‐induced mammary tumor metastasis could be reduced both by Cox2 inhibitors and EP4 antagonists in tumor bearing mice may be highly significant (Fig. 5G, H). Inclusion of such agents in fractionated radiotherapy protocols could thus be desirable.

In summary therefore, our studies have indicated the inherent plasticity of mammary cancer cells and their ability to respond to genotoxic stress through the transient induction of cells with enhanced migratory and metastatic properties. These findings have discernable implications for fractionated radiotherapy if such resistant and metastatic cells were to accumulate progressively in the cancers of human patients with each dose. However, the changes we observed here appeared in other studies to be triggered in tandem with radiation‐induced activation of tumor immunogenicity and specific cell killing by cytotoxic T cells (Formenti and Demaria, 2012; Surace *et al*., 2015). Competition between the pro‐metastatic and immunogenic effects of radiation may therefore mold overall responses to treatment. Our demonstration that radiation‐induced migration and metastasis could be induced by secreted factors suggests that these may encompass a series of accessible therapeutic targets, of which targeting secreted eicosanoid products have been exemplified herein.

## 4 Experimental procedures

### 4.1 Cell maintenance, irradiation, and inhibitors

4T1 and MCF7 cells were purchased from *The American Type Culture Collection* and cultured in Dulbecco's modified of Eagle's medium (DMEM) with 10% heat‐inactivated fetal calf serum, 2 mm l‐glutamine, and 100 μg·mL^−1^ of both penicillin and streptomycin (Cellgro, Mediatech, Tewksbury, MA, USA) in a Heracell CO_2_ incubator at 37 °C and 5% CO_2_. For irradiation treatment, cells were plated in 100‐mm tissue culture plates overnight and irradiated at the required doses with a Cesium^137^ source at a dose rate of 1.06 Gy·min^−1^ or 2.29 Gy·min^−1^. Cisplatin was purchased from Cayman Chemical Company (Ann Arbor, MI, USA) (L161,982), and ibuprofen, doxorubicin, and celecoxib were from Sigma, Franklin, MA, USA. MMT mammary tumor cells were from mice doubly transgenic for human mucin 1 Ag and polyomavirus middle T (PyMT) oncogene (Xia *et al*., 2003). MMT mice were a kind gift from Sandra J. Gendler, Mayo Clinic, Scottsdale, AZ. Such MMT mice carrying the PyMT oncogene driven by the mouse mammary tumor virus long terminal repeat developed mammary carcinoma at a 100% incidence (Guy *et al*., 1992).

### 4.2 FACS and immunocytochemical (ICC) staining

Cells were lifted with Accutase (ThermoFisher Scientific, Franklin, MA, USA) and washed in phosphate‐buffered saline pH7.4 and then stained with Fixable Viability Dye eFluor 450 and antibody against Sca‐1 (eBioscience, Franklin, MA, USA). For indicated experiments, cells were further stained with anti‐CD44 mAb (eBioscience) using standard immunofluorescence staining method. The Sca‐1 single‐ or Sca‐1^+^CD44^+^ double‐positive cells were quantified by FACS (Gallios, Beckman Coulter, Pasadena, CA, USA) and analyzed with FlowJo or FACScan. Dead cells were omitted from marker quantification based upon viability dye staining. To examine Sca‐1 expression by ICC, cells (1 × 10^4^ per plate) were cultured in small culture plates for indicated days after radiation treatment, cytocentrifuged onto the slide, and then stained with anti‐Sca‐1 antibody by standard ICC staining method and examined under light microscope. For Fig. S1A, cells were cultured on coverslips, fixed with 4% paraformaldehyde and permeabilized with 0.1% Triton‐×100. Cells were then stained with Sca‐1 antibody and mounted using ProLong Gold Antifade Mountant with DAPI (ThermoFisher Scientific).

### 4.3 Cell migration assays

Multiwell cell migration assays were performed following established protocol (Kouspou and Price, 2011). Multiwell chambers were incubated for the indicated time upon which cells on the upper side of filter were removed and those underside were then fixed and stained with 0.5% crystal violet staining or Sca‐1 antibodies. Cells were counted using 40× magnification.

### 4.4 Western blotting

Cell lysates were collected in RIPA buffer (Boston BioProducts, Ashland, MA, USA) containing Halt™ Protease and Phosphatase Inhibitor Cocktail (Thermo Scientific, Orlando, FL, USA). Protein content of samples was derived using Pierce™ BCA Protein Assay Kit (Thermo Scientific). Equal protein from cell lysates wase subjected to SDS/PAGE followed by established western blot procedure (Chou *et al*., 2015). Primary and secondary antibodies listed in Supporting information. The Ag/Ab signals were quantified with ECL reagent (GE) and autoradiography film (HyBlot CL, Denville Scientific, Holliston, MA, USA) or LI‐COR Odyssey (Lincoln, MA, USA). Immobilon‐FL and Immobilon‐P poly(vinylidene difluoride) membranes (Merck Millipore, Burlington, MA, USA) were used for near‐infrared (Odyssey) and HRP (HyBlot CL) detection methods, respectively. Densitometry of the membranes was performed using Image Studio Lite (LI‐COR Biosciences). imagej was used to adjust contrast of western blot figure images without disturbing the relative ratio of signal.

### 4.5 RT‐qPCR

Total RNA was harvested 24 h following the indicated treatment using RNeasy Mini Kit (Qiagen, Hilden, Germany) and converted to cDNA with iScript cDNA Synthesis Kit (Bio‐Rad, Hercules, CA, USA). Quantitative PCR amplification of 10 ng cDNA was performed using SYBR Green PCR Master Mix and 7300 Real Time PCR System (Thermo Fisher Scientific, Asheville, NC, USA) with cycle conditions: 50 °C 2 min, 95 °C 10 min, [95 °C 15 s, 60 °C 1 min] × 40 cycles. Dissociation curve analysis confirmed single PCR products. Amplicon amplification efficiency and *C*_t_ values were determined using the linregpcr software (Ruijter *et al*., 2009). Amplicon primer sequences are provided in the Supporting information with corresponding PrimerBank ID where appropriate (Wang, 2003).

### 4.6 PGE2 quantification by ELISA

Samples of radiation‐treated cell‐conditioned media and control media were collected and frozen at −80 °C until analysis. Media samples from doxorubicin‐treated cells were collected by replacing media on cells treated with doxorubicin for 48 h with agent‐free growth media and culturing cells for a further 6 h at which time the media were collected and stored at −80 °C. Cells were lifted and counted for normalization of PGE2 to sample cell number. Prior to ELISA assay, the cultured media samples were thawed on ice and clarified by centrifugation at 15 000 g for 15′ at 4 °C. PGE2 levels were quantified using Prostaglandin E2 ELISA Kit – Monoclonal (Cayman, Ann Arbor, MI, USA) as per the manufacturer's protocol.

### 4.7 Tumorsphere assay

Tumorsphere procedure was performed using methods previously published (Chang *et al*., 2012; Grange *et al*., 2008; Kim *et al*., 2010; Lee *et al*., 2015). 4T1 cells were treated as indicated, 24 h after second irradiation, cells were lifted using trypsin or accutase and strained through a 40‐μm cell strainer (USA Scientific, Orlando, FL, USA) and plated in 24‐well ultra‐low attachment plates (Sigma‐Aldrich, Natick, MA, USA) at 8–10 cells·μL^−1^ in tumorsphere culture medium. Tumorsphere culture medium consisted of DMEM/F12 medium with HEPES buffer, 10 ng·mL^−1^ epidermal growth factor, 10 ng·mL^−1^ basic fibroblast growth factor (Thermo Fisher Scientific), 4 μg·mL^−1^ heparin, 0.5% methylcellulose, 5 μg·mL^−1^ insulin (Sigma‐Aldrich, Natick, MA, USA), 0.4% bovine serum albumin (Thermo Fisher Scientific, Asheville, NC, USA), and 1 μm hydrocortisone (Stem Cell Technologies, Vancouver, BC, Canada). Tumorspheres were incubated at 37 °C and 5% CO_2_ for 7 days at which time they were imaged and counted using a Celigo High Throughput Micro‐Well Image Cytometer (Nexcelom Bioscience, Lawrence, MA, USA). Tumorsphere counts were performed using the Celigo tumorsphere mask analysis setting with parameters selected such that the minimum tumorsphere diameter was over 100 μm. General settings: analysis resolution, 4 μm per pixel; well mask, 96%; well mask usage automatic. Identification settings: colony diameter, 400 μm; precision, low; border dilation, 0; minimum thickness 0; background correction, yes. Prefiltering settings: area, 1 × 10^4^ to 2 × 10^6^ μm; intensity range, 0 to 255; min. aspect ratio, 0.05.

### 4.8 Assessment of ALDH activity

Cells at 1 × 10^4 ^cells·mL^−1^ were seeded in 100‐mm cell culture plates and irradiated with 6 Gy every second day up to three irradiations. The ALDH activity of the cells was assayed according by the ALDEFLUOR™ assay according to the manufacturer's instructions (Stem Cell Technologies). Briefly, 10^6^ cells were resuspended in 1 mL of aldefluor assay buffer with activated aldefluor substrate. As a negative control for each sample, an aliquot of ‘aldefluor‐exposed’ cells were immediately quenched with a specific ALDH inhibitor, DEAB. Following a 45′ incubation at 4 °C, the cells were centrifuged. Cell pellets were resuspended in 0.5 mL aldefluor assay buffer and analyzed by FACS (Becton Dickinson, Franklin Lakes, NJ, USA). For triple staining, suspended single cells were stained with PE‐conjugated Sca‐1 and Cy‐conjugated CD44 mAbs, and then stained for ALDH^+^ cells as described above. The ALDH^+^Sca‐1^+^CD44^+^ cells were analyzed by flow cytometry.

### 4.9 Radiotherapy *in vivo*

GFP^+^ MMT tumor cells (10^6^) were inoculated into the right and left mammary fat pads of wild‐type C57BL/6 mice. On day 10, the tumors (about 5 mm^3^) in the right side received radiation at 9 Gy using XRAD320 (Precision X‐Ray Inc., Branford, CT, USA). The mice were sacrificed on days 3 and 5 after radiation. Then sections of tumor or draining lymph node were prepared, stained with anti‐Sca‐1 antibodies, and examined by light microscopy. Mice were housed and handled in facilities accredited by the American Association for the Accreditation of Laboratory Animal Care. The study was carried out in accordance with the animal protocol approved by the Institutional Animal Care and Use Committee of Boston University Medical Center. The mice were sedated with intraperitoneal administration of ketamine (75 mg·kg^−1^) and xylazine (5 mg·kg^−1^), and then, the tumor was irradiated with 9 Gy by XRAD 320 (Precision X‐Ray Inc., N. Branford, CT, USA). The XRAD320 is a self‐contained X‐ray system for delivering a precise radiation dosage to the tumor in small animals. Thus, the systemic exposure of radiation was avoided. The mice were closely followed and would be euthanized if they showed inactivity, ruffled fur coat, or anorexia.

### 4.10 Statistical analysis

Statistical significance was determined using Student's *t*‐test or *X* ^2^‐test. One‐way analysis of variance (ANOVA) was used for analysis of data with more than two subgroups and Tukey's *post hoc* test was used for further statistical inference between subgroups. *P*‐values of < 0.05 were considered statistically significant. spss statistics software (IBM, Armonk, NY, USA) and Microsoft Excel were used to attain these values.

## Author contributions

JG, BJL, DW, TE, AM, TJB, SD, and BS devised and carried out the experimentation. JG, BJL, MAS, and SKC wrote the manuscript.

## Supporting information


**Fig. S1.** Sca‐1 expression is induced by genotoxic agents doxorubicin and celecoxib in 4T1 cells.
**Fig. S2.** Increased numbers of 4T1 Sca‐1^+^ cells after 6 Gy × 2 irradiation are not due to higher rates of Sca‐1^−^ cell death.
**Fig. S3.** Gene expression profiles of irradiated or non‐irradiated 4T1 tumor cells by qPCR array.
**Fig. S4.** ALDH activity in 4T1 and MCF7 cells.
