# Supplementary material for: Genotoxic stress induces Sca‐1‐expressing metastatic mammary cancer cells
Source: Mol Oncol. 2018 Jun 13;12(8):1249–63. doi: 10.1002/1878-0261.12321 (PMC6068352; doi:10.1002/1878-0261.12321)
Supplement: Supplementary file 1 — Fig. S1. Sca‐1 expression is induced by genotoxic agents doxorubicin and celecoxib in 4T1 cells. Fig. S2. Increased numbers of 4T1 Sca‐1+ cells after 6 Gy × 2 irradiation are not due to higher rates of Sca‐1− cell death. Fig. S3. Gene expression profiles of irradiated or non‐irradiated 4T1 tumor cells by qPCR array. Fig. S4. ALDH activity in 4T1 and MCF7 cells. [file MOL2-12-1249-s001.pdf]

Supplementary Materials:

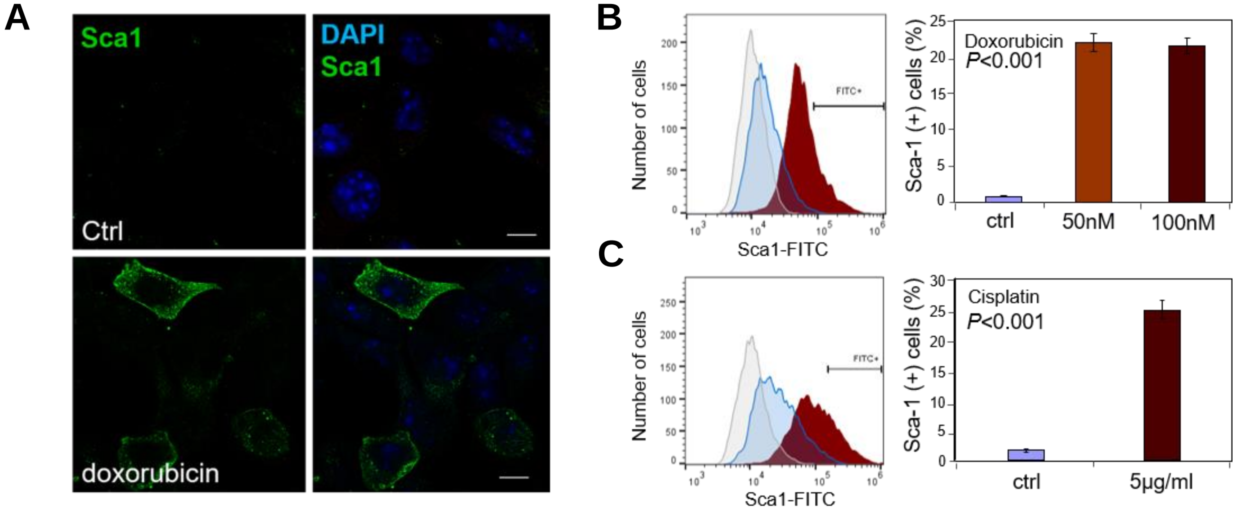

Suppl. Fig. 1. Sca-1 expression is induced by genotoxic agents doxorubicin and celecoxib in 4T1 cells (A) 4T1 cells treated with doxorubicin 100nM for 48 h were immuno-stained for Sca-1, scale bar 10µm, n=2. (B) Sca-1 expression of 4T1 cells treated with doxorubicin 100nM for 48 h measured by flow cytometry. Gray-IgG ctrl, blue-non-treated 4T1, maroon- 100nM doxorubicin. Graph represents mean Sca-1$^+$ cells ± SD for one representative experiment, n=4. (C) Sca-1 expression of 4T1 cells treated with cisplatin 5µg/ml for 48 h measured by flow cytometry. Graph represents mean Sca-1$^+$ cells ± SD for one representative experiment, n=3.

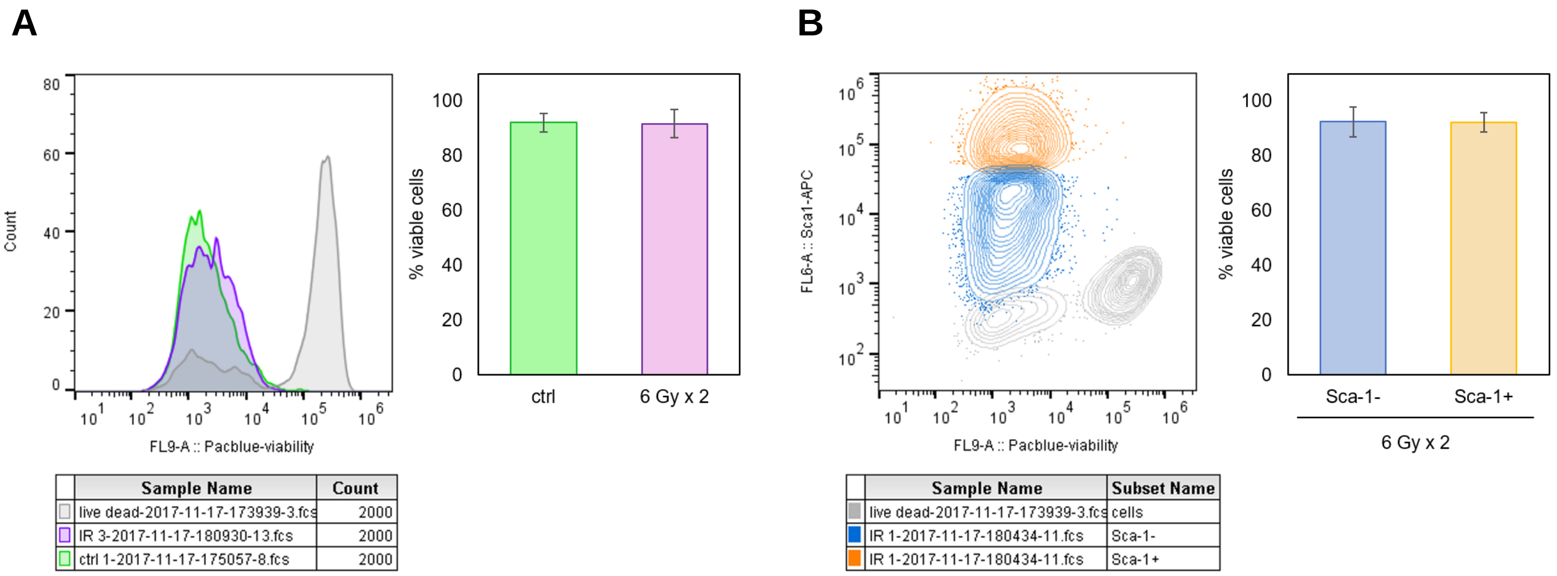

Suppl. Fig. 2. Increased numbers of 4T1 Sca-1^{+} cells after 6 Gy x 2 irradiation are not due to higher rates of Sca-1^{-} cell death. (A) Left; representative histogram of Pacblue viability signal assessed by flow cytometry of non-irradiated control 4T1 (green) and 6 Gy x 2 irradiated (purple) 4T1 cells after 24 h recovery. Control 4T1 cells mixed with snap-frozen - thawed 4T1 cells acted as a positive control for alive/dead staining (Gray). Right; The mean percentage across three independent experiments of alive cells within irradiated (6 Gy x 2) 4T1 and non-irradiated 4T1 cells is shown ± SEM. (B) Left; representative contour plot of Pacblue viability signal of irradiated (6 Gy x 2) 4T1 cells stained with anti-Sca-1-FITC antibody (y-axis); Sca-1^{+} (orange) and Sca-1^{-} (blue) populations are shown. Snap-frozen and thawed 4T1 cells mixed with untreated cells acted as a positive control for alive and dead cells (Gray). Right; The mean percentage of alive cells within irradiated (6 Gy x 2) 4T1 Sca-1^{+} and Sca-1^{-} populations across three independent experiments is shown ± SEM.

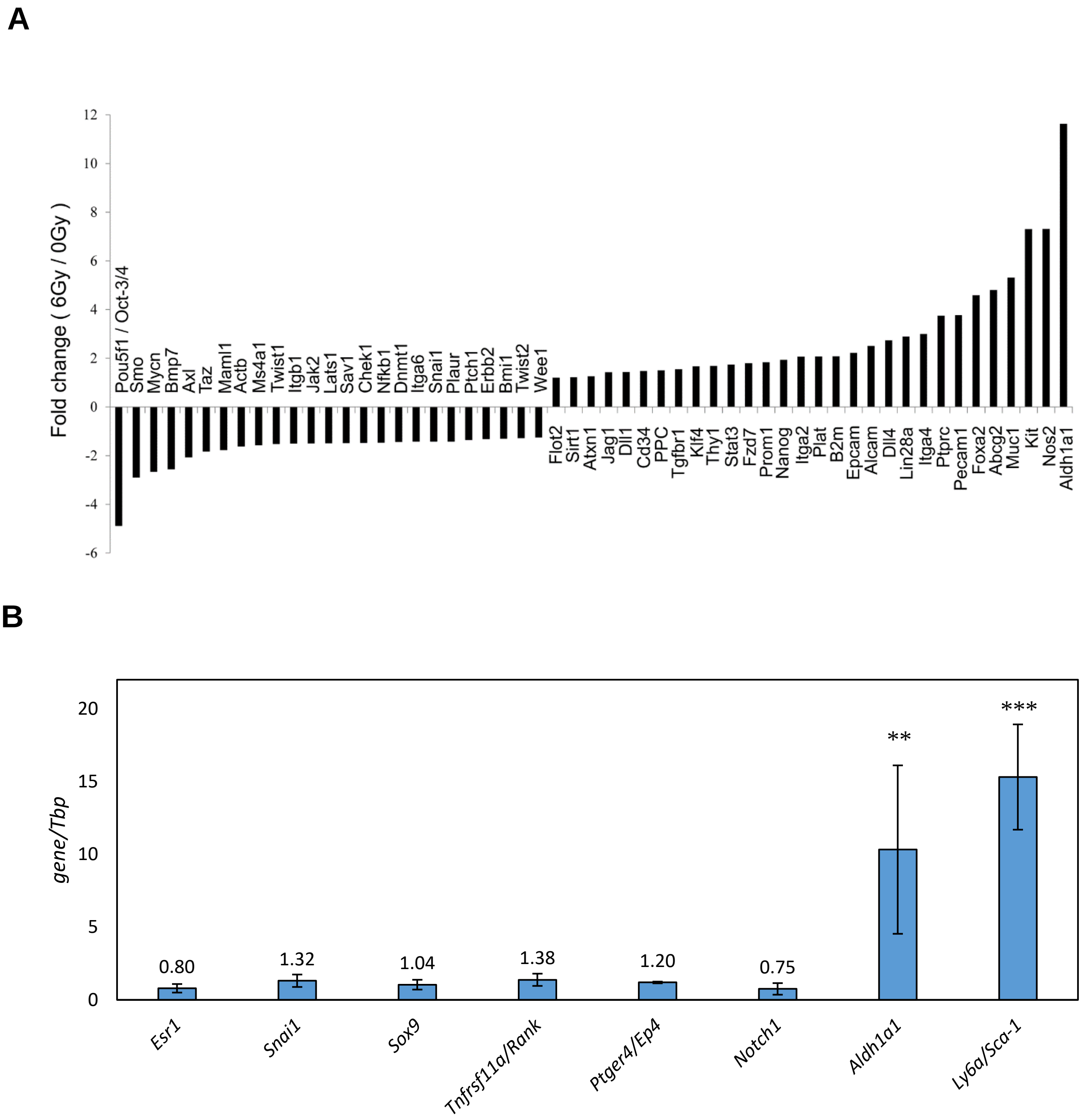

Suppl. Fig. 3. Gene expression profiles of irradiated or non-irradiated 4T1 tumor cells by qPCR array. (A) PCR array (mouse CSC array) was used to detect the gene expression between irradiated and non-irradiated 4T1 tumor cells. The mRNAs extracted from 4T1-0Gy and 4T1-6Gy tumor cells were assayed by the RT^2 -qPCR array for stem cells-associated transcripts. The up- or down-regulated genes with twice or more fold changes were presented (B) RT-qPCR expression analysis of indicated genes in 4T1 cells irradiated 6 Gy x 3 + 24 h recovery. Mean gene expression relative to non-irradiated control for at least three independent experiments is shown ± SEM, ** P <0.01 for each repeated experiment, *** P <0.001 for each repeated experiment.

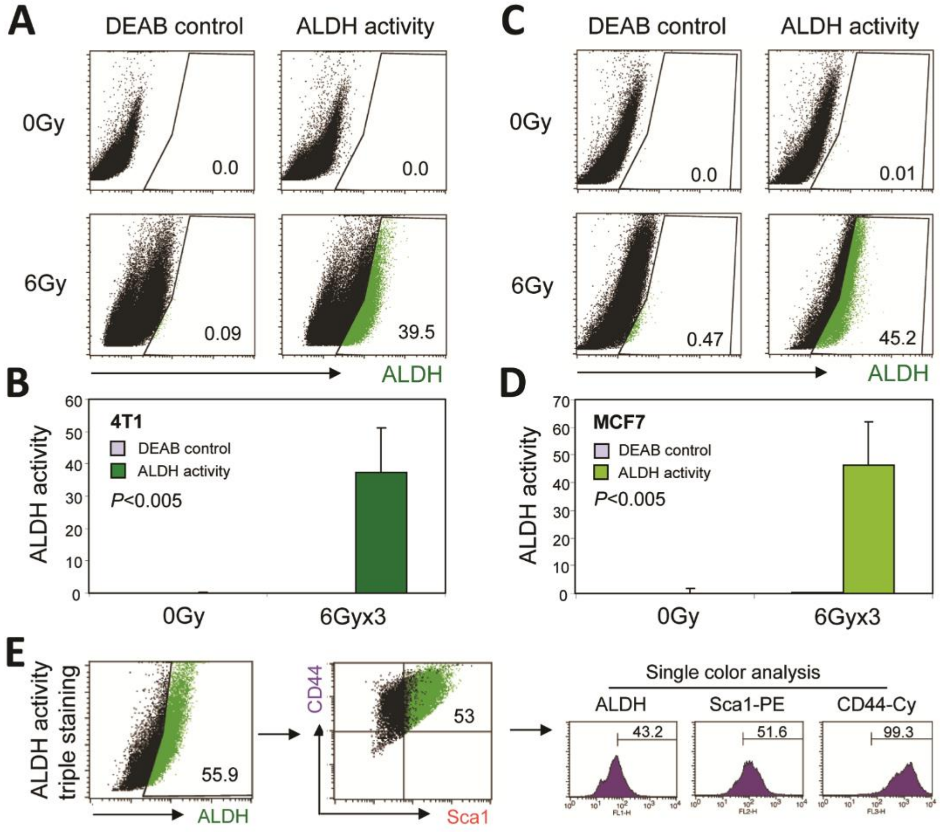

Suppl. Fig. 4. ALDH activity in 4T1 and MCF7 cells. An ALDEFLUOR™ assay was used to detect the ALDH activity in 4T1 cells (A) and MCF7 (C) cells with or without irradiation. The ALDEFLUOR™ DEAB-treated groups in each sample were used as negative control. The cell population in each group with green color in dot plots indicate positive for ALDH. (B and D) The percentage of 4T1 and MCF7 cells positive for ALDH in three experiments was presented in bar graph. The error bars indicate standard deviation and the *P* value is less than 0.005. (E) Detection of Sca-1^{+}CD44^{+}ALDH1^{+} 4T1 cells induced by IR. The 4T1 cells treated without or with three times 6Gy radiation were stained with Sca1-PE and CD44-Cy and then subjected to ALDEFLUOR™ assay to show ALDH positive cells. The cells were analyzed by flow cytometry. The cells with green color were ALDH positive cells (left panel) (FL1) (G1 gate). The triple positive cells were shown in the middle panel (G1 gated cells with Sca-1-PE (FL2) and CD44 –Cy (FL3). The percentage of ALDH, Sca-1 and CD44 positive cells was shown in the histogram (right panel).

Supplemental Experimental Procedures

qPCR array

For PCR Array, 5µg of total RNA was used. Procedures involved genomic DNA elimination followed by reverse transcription carried out using an RT^2 first strand kit (Qiagen). An RT^2 profiler PCR array for mouse cancer stem cells (Qiagen) was used for gene expression profiling. 84 genes including 5 housekeeping genes were analyzed in control (0Gy 4T1 samples) and after 6Gy. Threshold for analysis of Ct value were adjusted between samples based on Ct in housekeeping genes.

RT-qPCR primers			
Gene symbol	Forward	Reverse	Primerbank ID
Ptgs2	CAGCCAGGCAGCAAATCCTT	GGGTGGGCTTCAGCAGTAAT	
Tbp	GGCCTCTCAGAAGCATCACTA	GCCAAGCCCTGAGCATAA	
Ly6a/Sca1	AGGAGGCAGCAGTTATTGTGG	CGTTGACCTTAGTACCCAGGA	6754580a1
Aldh1a1	ATACTTGTCGGATTTAGGAGGCT	GGGCCTATCTTCCAAATGAACA	7304881a1
Notch1	CCCTTGCTCTGCCTAACGC	GGAGTCCTGGCATCGTTGG	31543332a1
Ptger4/Ep4	ACCATTCCTAGATCGAACCGT	CACCACCCCGAAGATGAACAT	6679531a1
Tnfrsf11a/Rank	CAGCATCGCTCTGTTCCTGTA	CTGCGTTTTCATGGAGTCTCA	6755833a1
Sox9	CGGAACAGACTCACATCTCTCC	GCTTGCACGTCGGTTTTGG	165932320c2
Snai1	CACACGCTGCCTTGTGTCT	GGTCAGCAAAAGCACGGTT	6755586a1
Esr1	CCTCCCGCCTTCTACAGGT	CACACGGCACAGTAGCGAG	6679695a1

Antibodies for Western blot		
antigen	catalog number	source
Cox2	12282	Cell Signaling Technologies
β-actin	A5441	Sigma
anti-rabbit IgG HRP	ab6721	Abcam
anti-mouse IgG HRP	ab6721	Abcam
anti-rabbit IgG 800CW	926-32211	LI-COR
anti-mouse IgG 680RD	926-68070	LI-COR

FACS antibodies		
Sca1 clone D7	11-5981-82	eBioscience
CD44 clone IM7	15-0441	eBioscience
